# Drug Repurposing for Cancer Treatment: A Comprehensive Review

**DOI:** 10.3390/ijms252212441

**Published:** 2024-11-19

**Authors:** Abdulaziz H. Al Khzem, Mohamed S. Gomaa, Mansour S. Alturki, Nada Tawfeeq, Mohammad Sarafroz, Shareefa M. Alonaizi, Alhassan Al Faran, Laela Ahmed Alrumaihi, Fatimah Ahmed Alansari, Abdullah Abbas Alghamdi

**Affiliations:** 1 Department of Pharmaceutical Chemistry, College of Clinical Pharmacy, Imam Abdulrahman Bin Faisal University, P.O. Box 1982, Dammam 31441, Eastern Province, Saudi Arabia; msalturki@iau.edu.sa (M.S.A.); nztawfeeq@iau.edu.sa (N.T.); mskausar@iau.edu.sa (M.S.); 2 College of Clinical Pharmacy, Imam Abdulrahman Bin Faisal University, P.O. Box 1982, Dammam 31441, Eastern Province, Saudi Arabia; 2200002503@iau.edu.sa (S.M.A.); alfaran32@gmail.com (A.A.F.); 2210000333@iau.edu.sa (L.A.A.); 2220001110@iau.edu.sa (F.A.A.); abdullahabs2002@gmail.com (A.A.A.)

**Keywords:** repurposing, anticancer, drug discovery, therapeutic classes, translational research, drug targets

## Abstract

Cancer ranks among the primary contributors to global mortality. In 2022, the global incidence of new cancer cases reached about 20 million, while the number of cancer-related fatalities reached 9.7 million. In Saudi Arabia, there were 13,399 deaths caused by cancer and 28,113 newly diagnosed cases of cancer. Drug repurposing is a drug discovery strategy that has gained special attention and implementation to enhance the process of drug development due to its time- and money-saving effect. It involves repositioning existing medications to new clinical applications. Cancer treatment is a therapeutic area where drug repurposing has shown the most prominent impact. This review presents a compilation of medications that have been repurposed for the treatment of various types of cancers. It describes the initial therapeutic and pharmacological classes of the repurposed drugs and their new applications and mechanisms of action in cancer treatment. The review reports on drugs from various pharmacological classes that have been successfully repurposed for cancer treatment, including approved ones and those in clinical trials and preclinical development. It stratifies drugs based on their anticancer repurpose as multi-type, type-specific, and mechanism-directed, and according to their pharmacological classes. The review also reflects on the future potential that drug repurposing has in the clinical development of novel anticancer therapies.

## 1. Introduction

Cancer is a collection of multiple diseases that occur progressively due to uncontrollable cell proliferation [1,2]. Each type has unique characteristics, but all share fundamental processes contributing to the disease’s development [3,4]. Cancer cells can be malignant or normal, and they expand when not provided with signals, disregarding signals to cease proliferation or undergo apoptosis. They direct vascular proliferation toward malignancies, providing oxygen and nutrition while eliminating toxic materials. They also avoid the immune system’s attention, facilitating their proliferation and survival. Cancer cells often have a diverse array of chromosomal modifications, which they rely on to the extent that they cannot function normally [5].

Tumor progression is typically illustrated through stages of mutation and growth. A normal cell is transformed into a malignant cell with less than 10 mutations [2,6]. Stages include initial mutation, hyperplasia, dysplasia, in situ cancer, and invasive/malignant tumors. In situ cancer is characterized by abnormal development and appearance of the cell and its progeny, while invasive/malignant tumors allow the tumor to disseminate to other tissues and discharge cells into the lymph or bloodstream, potentially generating new malignancies.

Malignant tumors can spread throughout the body, causing resistance to targeted therapy [3]. Cancer-critical genes are categorized into two primary categories: proto-oncogenes and tumor suppressor genes [7]. Proto-oncogenes facilitate cell proliferation, while tumor suppressor genes prevent it. Mutations in these genes can lead to the overactivity of proteins involved in growth-promoting pathways, causing cells to multiply faster than they would without mutation [2].

Most malignancies are classified into three main categories: carcinomas, sarcomas, and leukemias or lymphomas. Carcinomas account for 90% of human cancers, while sarcomas are solid tumors affecting connective tissues. Lymphomas and leukemias are caused by the immune system and blood-forming cells, accounting for 8% of all human malignancies. Tumors are further classified by cell type and tissue of origin [8].

**Figure 1:**
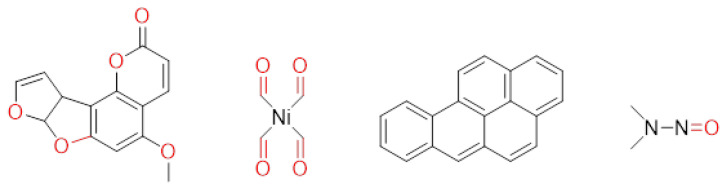
Illustration of chemical carcinogens that induce DNA damage and mutations.

Mutations and DNA damage are induced by radiation and chemical carcinogens, which are considered “starting agents” because mutations in critical target genes are the earliest event leading to cancer formation (Figure 1). Carcinogenic chemicals in tobacco smoke, sunlight ultraviolet radiation, and aflatoxin are the primary initiating agents contributing to human cancers. Smoking is responsible for 80 to 90% of lung malignancies and is associated with other body regions [9].

Hormones, especially estrogens, can promote certain human malignancies, such as endometrial cancer and breast cancer. Excessive estrogen exposure increases the risk, while progesterone therapy can mitigate this risk. Prolonged use of estrogen and progesterone combinations can also increase breast cancer risk [10].

### 1.1. Cancer Statistics for Saudi Arabia

In 2022, Saudi Arabia experienced 13,399 cancer-related fatalities and 28,113 new cancer cases. Breast cancer, colon–rectum cancer (CRC), and thyroid cancer comprise the most prevalent malignancies. Hormonal variations, diet, lifestyle, and obesity were the most frequently reported risk factors associated with breast cancer. A total of 13.3% of all malignancies in the nation were attributed to CRC in 2022. Genetic, environmental, age, gender, and other inflammatory conditions of the digestive tract may all be risk factors for CRC. Thyroid carcinoma is a thyroid disorder that is frequently diagnosed, with a prevalence of 9.3%. Goitre disease, exposure to excessive levels of radiation, and family history are all risk factors for thyroid cancer [11].

In Saudi Arabia, the age-standardized rates (ASR) indicate that the incidence of all malignancies is 87.1, with ASR incidence rates of 81.0 in males and 100.4 in females. The estimated mortality rate was 13,399 cases, regardless of gender or age. The mortality rates of ASR were 48.4 in males and 45.0 in females. Among all Arabian Gulf countries, Saudi Arabia has the second-highest cancer mortality rate [12].

In Saudi Arabia, breast cancer ranked first among other cancer types, with a mortality rate of 7.6% and an incidence rate of 25.3% for both genders. The incidence of breast cancer among females was 25.3% in 2022 [13].

Colorectal cancer was the second most prevalent form of cancer in Saudi Arabia, with an incidence of 11.7% and a mortality rate of 6.2% for both genders. Males experienced a 13% incidence rate, while females experienced a 9.8% incidence rate [14].

**Figure 2:**
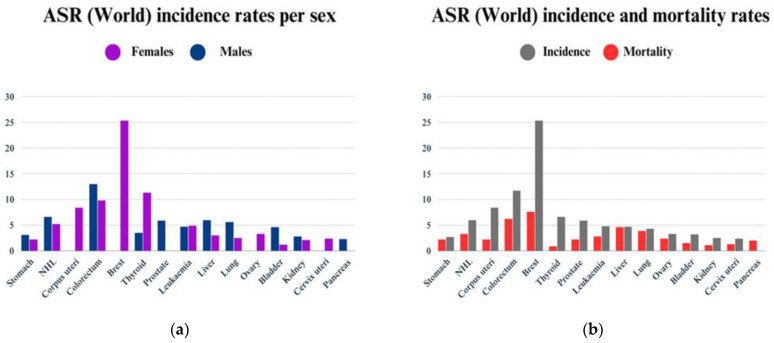
(**a**) ASR incidence rates per sex; (**b**) ASR incidence and mortality rates.

Thyroid cancer was the third most prevalent form of cancer in Saudi Arabia, with an incidence of 6.6% and a mortality rate of 0.9% for both genders. The incidence was significantly higher among females at 11.3% than among males at 3.5%, as illustrated in Figure 2 [15].

### 1.2. Challenges Associated with the Development of Anticancer Drugs

The ideal approach in pharmacotherapy involves medications that precisely target and eliminate malignant cells while minimizing side effects. However, significant differences between normal and cancerous cells, such as variations in signaling pathway activation, hormone or growth factor sensitivity, and growth behaviors, complicate the development of effective cancer treatments. Despite these challenges, advancements in effective medications, interdisciplinary and personalized treatments, and enhanced palliative care services, including targeted therapies and potent chemotherapeutic agents, have contributed to improved patient survival rates [16].

Despite these advancements, numerous treatment failures persist, primarily due to various drug resistance mechanisms. Additionally, the cost of new cancer therapies has significantly risen due to extensive pharmacological research. This financial burden is becoming increasingly difficult for most healthcare systems to sustain, necessitating innovative approaches to repurpose and improve existing medications [16].

The cost of cancer care surged by approximately 19%, 31%, and 28% in 2020 for the initial, ongoing, and final phases of therapy, respectively, compared to 2010 [16]. Another significant challenge in oncologic research is the complexity of cancer, which involves numerous molecular patterns with varying susceptibilities and resistances to different therapies. Therefore, developing more effective cancer treatments is crucial [16].

### 1.3. Drug Repurposing

Drug repurposing is one strategy under extensive investigation and implementation to tackle this issue [4,17,18,19,20,21]. Drug repurposing is the use of an already-approved medication for a new medical condition or treatment, often leveraging unexpected side effects or unknown therapeutic effects [22,23]. This approach saves time and money in pharmaceutical research by bypassing the traditional drug development pathway and transitioning to preclinical and clinical trials [24]. Traditional drug development is known for its lengthy timelines and high costs, often requiring at least USD 1 billion and ten years to bring a new medication to the market [25]. Drug repurposing focuses on quickly identifying existing medications that can effectively treat conditions beyond their original intended use, particularly when traditional de novo drug development is impractical or when urgent therapeutic solutions are needed [26]. There are several methods of drug repurposing, including developing approved drugs for new indications, repurposing approved and marketed molecules for off-label use through clinical testing, and rescuing molecules abandoned due to issues like poor efficacy or toxicity [27]. Regulatory bodies like the FDA attribute a substantial proportion of newly approved medications and biologics to drug repurposing [28].

The application of drug repurposing in cancer therapy provides better mitigation options for cancer patients faster and at a lower cost by repositioning non-oncology medications to combat cancer cells. Various techniques were employed to investigate the potential anticancer effects of non-cancerous medications. Numerous in vivo and in vitro studies using cancer cell lines and pharmacological models were conducted to explore the drug repurposing of non-oncology drugs. Several comprehensive electronic databases, including the Molecular Libraries Initiative and the National Institutes of Health (NIH), have been used in the analysis of chemical compounds, biological assays, and the genetic significance of active chemical compounds in the context of drug repurposing.

Repurposing non-oncology drugs could be at any level of the cancer pathophysiological mechanisms, including induction of apoptosis, inhibition of growth signaling and transducers, inhibiting cancer cell metabolism, activation of antitumor immunity, reactivation of growth suppressors, interference with replication, reduction in tumor blood supply, and suppression of invasion and metastasis [29,30,31,32]. However, drug repurposing still has its shortcomings, including intellectual property barriers that may hinder further development and the chance of failing the higher phases of clinical trials due to lower efficacy compared to current therapy or potential toxicity for the relevant clinical trial design.

## 2. Drug Repurposing Cases for Cancer Treatment

This section will outline medications that have been repurposed in cancer treatment to the best of our knowledge and report on their current status. A summary is provided in Table 1, Table 2 and Table 3.

### 2.1. Repurposing with a Multi-Type Anticancer Activity

#### 2.1.1. Repurposing Anti-Platelet Medication for Cancer Treatment

Aspirin, initially used for cardiovascular conditions, has shown anti-tumoral activity and is now being repurposed for treating triple-negative breast cancer. Studies demonstrated that regular aspirin use is associated with a lower risk of breast cancer. Aspirin has been found to inhibit the growth of breast cancer cells with PIK3CA mutations by activating signaling pathways that inhibit mTORC1 and AMPK. Additionally, variations in PIK3CA SNPs have been linked to different outcomes of aspirin therapy in breast cancer patients. As a result, authors [33] proposed that combining PI3K inhibitors with aspirin could be an effective treatment strategy for breast cancer, provided patients are stratified based on their genetic profile concerning the PI3K gene.

Aspirin inhibits cyclooxygenases (COXs), which are enzymes involved in the production of prostaglandins and thromboxane, chemical messengers that contribute to pain signaling, blood clotting, and inflammation. By reducing inflammation, aspirin may help prevent cancer development and progression. Additionally, aspirin may enhance anti-tumor immune activity and support other anticancer immunotherapies by reducing platelet activity. Studies have shown that aspirin can inhibit various tumor cell activities and disrupt the tumor microenvironment, hindering cancer cell growth and spread [34,35,36,37,38,39].

Low-dose aspirin use has been linked to a reduced risk of various cancers, including gastric, esophageal, colorectal, pancreatic, ovarian, endometrial, breast, and prostate cancers. However, a meta-analysis found no significant difference in cancer incidence or mortality between aspirin users and non-users. Regular low-dose aspirin use has also been associated with improved survival rates, with studies showing a lower 10-year risk of cancer and total mortality compared to non-users. After a cancer diagnosis, low-dose aspirin use has been associated with lower mortality in patients with digestive tract cancers. A meta-analysis of eight studies found a strong correlation between aspirin use and a reduced risk of hepatocellular carcinoma, the most common type of primary liver cancer. Low-dose aspirin use is also associated with a 10% lower risk of developing ovarian cancer. However, the relationship between aspirin use and survival in ovarian cancer patients has not been thoroughly studied [32,40].

#### 2.1.2. Repurposing Anti-Diabetic Medication for Cancer Treatment

Several studies have documented the benefits of anti-diabetic medications in cancer management. Anti-diabetic drugs from various pharmacological classes and different chemical groups, including the classic groups, sulfonylureas, thiazolidinediones, biguanides, as well as the recent groups, sodium-glucose co-transporter-2 (SGLT2) inhibitors, and dipeptidyl-peptidase IV inhibitors, are effective in treating certain cancers. The metabolic links between diabetes and cancer, including the direct effects, hyperglycemia, hyperinsulinemia, and the indirect effects, inflammation, oxidative stress, and obesity, are thought to contribute to the anticancer effects of these drugs.

Recent studies have demonstrated that anti-diabetic drugs can decrease the occurrence of cancer by directly impacting the metabolism of cancer cells and indirectly by modifying markers associated with the risk of tumor development. Special attention was given to insulin sensitizers, including metformin and the thiazolidinedione group, due to their impressive results in clinical trials. Metformin is the principal treatment for type 2 diabetes and is widely recognized as the most prominent example. Multiple meta-analyses of case-control, cohort studies, observational, and clinical trials have provided evidence of its efficacy in cancer prevention and treatment, whether used alone or in conjunction with other drug therapies. Metformin substantially lowers the likelihood of developing colorectal, breast, pancreatic, prostate, lung, and cervical malignancies. Research suggests that using biguanides is associated with a 20–30% reduction in the incidence of all cancers and cancer-related deaths [41,42]. Thirty-seven clinical trials have assessed metformin as a stand-alone treatment or in combination with conventional therapy. Its efficacy against colon cancer has progressed to Phase II and III clinical trials when used alone before and during surgery and in combination with conventional therapy. Metformin induces cell cycle arrest in various colon cell lines through specific mechanisms [43,44,45,46]. Metformin is particularly effective in preventing breast cancer, with over seven clinical trials currently evaluating its potential as a breast cancer treatment. It has progressed to Phase III when used alone or in combination with atorvastatin and to Phase II when combined with chemotherapeutic drugs, diet, or fasting. Metformin suppresses cancer stem cells (CSCs) in vitro by down-regulating CSC-specific genes and re-expressing miRNAs. It also decreases pSTAT3 phosphorylation, inhibits the activity of S6 kinase and mTOR, increases AMPK, and reduces mRNA translation in breast cancer. Studies have demonstrated that metformin effectively prevents skin cancer through several mechanisms. For example, metformin inhibits the production of TRB3 (tribbles pseudo kinase 3) in vivo, halting melanoma cells in the G0/G1 phase and reducing melanoma growth and spread. Additionally, metformin activates AMPK, affecting the tumor microenvironment and melanoma cell death and proliferation. In various melanoma cell lines, metformin also reduces cell proliferation and invasion by modulating the miR-192-5p/EFEMP1 and miR-584 3p/SCAMP3 axis [47,48,49].

Both preclinical and clinical research have demonstrated the potential of thiazolidinediones (TZDs) in the treatment of breast and prostate cancer. The primary constituents of this category are troglitazone, rosiglitazone, and pioglitazone. These compounds exert their anticancer actions by both PPAR γ (peroxisome-proliferator-activated receptor gamma)-dependent and independent pathways. TZDs stimulate the PPAR γ receptor, which then combines with the retinoid X-receptor to create a heterodimer. This interaction results in enhanced apoptosis and differentiation, as well as reduced cell population division. Targeted cytotoxic drugs (TZDs) depend on the expression of PTEN/AMPK, AKT/mTOR, and the breakdown of cyclins D1 and D3, regardless of PPAR γ [50,51].

While there are currently no other anti-diabetic therapies undergoing Phase II studies specifically for colon cancer, two pharmacological agents, desmopressin and pioglitazone, have demonstrated effectiveness against colon cancer cells by distinct methods. In vitro and in vivo, desmopressin, a selective agonist for vasopressin receptor 2 (AVPR2), suppresses the growth of colon cancer cells. By decreasing the expression of COX-2 and cyclin D1, pioglitazone, a synthetic PPAR ligand that enhances insulin sensitivity and glycemic management, inhibits metastasis of different colon cancer cells [30,52,53,54].

The mechanism by which pioglitazone induces G0/G1 arrest in MCF7 breast cancer cells, which is partly dependent on PPAR and partly dependent on MAPK, has not been investigated in clinical trials for breast cancer therapy [55,56,57,58].

Two anti-diabetic drugs are now undergoing clinical studies to determine their efficacy in preventing pancreatic cancer. Current Phase II trials are studying the use of pioglitazone in conjunction with conventional medication, while Phase I trials are investigating the use of metformin in combination with digoxin and simvastatin. Administering pioglitazone alone enhances the production of carcinoembryonic antigen mRNA both in laboratory settings and in living organisms. Furthermore, metformin by itself demonstrates encouraging inhibitory actions against several pancreatic cancer cells both in laboratory settings and in living organisms [59,60,61].

#### 2.1.3. Repurposing Anti-Helminthic Medication for Cancer Treatment

Anti-parasitic medications, such as chloroquine and ivermectin, as well as mebendazole, flubendazole, and albendazole, have shown potential as anticancer agents. These drugs, initially used to treat intestinal parasites, have demonstrated anticancer activity by targeting pathways like Wnt/β-catenin, NF-kB, signal transducer, and activator transcription proteins [62,63].

Flubendazole, a well-known benzimidazole, exhibits antineoplastic properties against various cancers, including neuroblastoma, multiple myeloma, leukemia, and breast cancer. It induces apoptosis, generation of reactive oxygen species, and activation of caspase 3 and 7. Flubendazole suppresses the proliferation of tumors and the formation of new blood vessels in lung, liver, and breast cancer by selectively attacking cancer cells, triggering cell death, and augmenting the expression of human epidermal growth factor receptor 2 (HER2) in breast cancer [64,65,66].

By inhibiting the autophagy pathway, signal transducer, and transcription protein 3, flubendazole exerts lethal effects in human colorectal cancer. It also inhibits melanoma cell proliferation and metastasis, leads to the accumulation of myeloid-derived suppressor cells, and induces programmed cell death protein-1 [67,68].

Benzimidazole-based anthelmintics have also shown in vitro and in vivo antitumor effects in various cancer models, including pancreatic cancer (PC). Parbendazole, administered at therapeutic plasma concentrations, induces apoptosis, disrupts the cell cycle, and triggers a DNA damage response, inhibiting PC cell growth and clonogenicity. Parbendazole significantly alters microtubule organization in PC cells, leading to rapid polyploid cell formation and aberrant mitotic spindles. The potential of parbendazole is further underscored by its synergistic effect with the standard PC chemotherapeutic drug gemcitabine. Bioinformatic tools identified several unexplored cancer-related targets for benzimidazoles, including the MAP kinase p38 alpha, VEGFR2, and tyrosine-protein kinase ABL, suggesting the need for further in vitro and in vivo studies in PC and other tumor models to validate these findings [52].

Mebendazole (MZ), commonly used to treat intestinal parasite infections, inhibits tubulin polymerization, leading to anti-parasitic effects. As a repositioning drug, mebendazole has shown synergistic effects with temozolomide in treating malignant gliomas, inhibiting tumor growth more effectively than temozolomide alone in both xenograft and syngeneic glioma models. Mebendazole also shows synergistic effects with docetaxel in blocking tubulin polymerization, inducing mitotic arrest in the G2/M phase, increasing apoptosis, and reducing prostate cancer cell proliferation and tumor growth. In vivo and in vitro studies demonstrate that mebendazole halts mitotic progression in the G2/M phase, induces double-strand breaks, and triggers apoptosis in breast cancer [69,70].

Additionally, mebendazole causes apoptosis by activating the caspase-3 pathway and prevents pulmonary metastases in advanced thyroid cancer. It also exhibits cytotoxicity in brain tumors and ovarian, colon, and endocrine cancers. Mebendazole induces apoptosis in cholangiocarcinoma by upregulating caspase 3, preventing cell division. Additional research indicates that mebendazole inhibits the growth of various cancer cell lines, including glioblastoma, medulloblastoma, melanoma, lung cancer, and colorectal cancer, both in vivo and ex vivo. Mebendazole induces apoptotic cell death in melanoma by activating caspases and the pro-apoptotic Bcl-2 while suppressing XIAP, the regulator of the apoptotic pathway [71].

Moreover, mebendazole exhibits a high affinity for colon cancer cells, indicating its potential role as a suppressor of Hedgehog signaling in medulloblastoma and as an inhibitor of kinases and oncogenes, such as ABL and BRAF. Mebendazole-treated rats show reduced tumor growth and angiogenesis, with a decreased incidence of metastases. Case studies indicate that therapeutic mebendazole administration can have anticancer effects, with one early study showing slowed lung cancer cell growth without affecting fibroblasts or normal endothelial cells. Patients with metastatic colorectal cancer received mebendazole after first- and second-line treatments, experiencing minimal side effects and significant reductions in pulmonary and lymphatic metastases, as well as partial hepatic metastasis recovery. Patients with adrenocortical carcinoma receiving mebendazole after unsuccessful chemotherapy had stable disease for about a year and a half, with no side effects and reduced metastase size [72].

Niclosamide, an FDA-approved anthelmintic drug, has been shown to interfere with anaerobic metabolism and glucose absorption in prostate, breast, and ovarian cancer cells. It also acts on increasing the transduction of transcription proteins Wnt/β-catenin and NF-KB, affecting various signaling pathways, including metastasis and signal activation. Niclosamide significantly slows the growth of colon, liver, and breast cancers and prevents colorectal cancer cells from metastasizing to the liver and breast cancer metastases to the lungs. However, its clinical development is hindered by poor solubility and lower bioavailability, which might be addressed by intravenous administration [73,74].

Clioquinol, another anti-parasitic drug, has demonstrated anticancer properties by down-regulating HDAC expression in leukemia and malignant myeloma cells. It induces apoptosis by down-regulating HDAC and causing mitotic arrest, leading to p53 and p21 expression [75,76].

#### 2.1.4. Repurposing Anti-Viral Medication for Cancer Treatment

Recognized for their anticancer properties, antiretroviral therapies (ARTs) can induce cell edema, apoptosis, and altered membrane permeability, leading to cell death. ARTs trigger apoptosis, inhibit angiogenesis, and enhance the effectiveness of chemotherapy or radiation by producing toxic free radicals through a ferrous iron-mediated reaction. Reactive oxygen species (ROS) generated by ARTs cause oxidative DNA damage and apoptosis, making cancer cells more vulnerable due to their lack of antioxidant enzymes. ARTs, particularly dihydroartemisinin, increase ROS before cytotoxic effects occur, indicating ROS as a primary cause of cell damage. ARTs target iron-rich cancer cells, enhancing their efficacy against tumor cells with high transferrin receptor (TfR) expression. Studies show that ARTs combined with amino acetic ferrous sulfate increase ART susceptibility in cancer cells, while deferoxamine mesylate pretreatment counteracts dihydroartemisinin-induced apoptosis in leukemia cells [77].

Protease inhibitors used in antiretroviral therapy, such as ritonavir, target HIV and have shown anticancer effects in ovarian, pancreatic, and breast cancers by triggering apoptosis and inhibiting cancer cell growth. Ritonavir acts synergistically with other drugs, including temozolomide in glioma cancer cells and bortezomib in kidney carcinoma. Ritonavir has demonstrated efficacy in treating lymphocytic leukemia and inhibits Akt phosphorylation in breast cancer cells [78,79,80].

Ribavirin is a guanosine ribonucleoside-based anti-viral drug that competes with guanylyl transferase and inhibits 5′-mRNA, reducing cyclins D1 protein levels and inhibiting oncogene transformation. Ribavirin also induces VEGF mRNA translation, suppressing human lymphocyte progression. Cidofovir, another FDA-approved anti-viral drug, inhibits viral DNA polymerase and treats various viral infections by blocking diphosphate metabolites. It activates caspase and PARP in epithelial cells, promoting apoptosis and mitotic arrest. Cidofovir inhibits DNA synthesis and cancer cell proliferation in glioblastoma cell lines. Both in vitro and xenograft models show cidofovir’s anti-proliferative properties, suppressing apoptosis-related genes in glioblastomas [81].

#### 2.1.5. Repurposing Cardiovascular Medications for Cancer Treatment

FDA-approved cardiovascular drugs have shown potential anticancer properties in various studies.

- Anti-Hypertension Medication

Losartan, an angiotensin receptor blocker (ARB), normalizes the tumor microenvironment by depleting the matrix and reducing collagen I levels, inhibiting tumor growth and cell proliferation. Losartan has shown promise in Phase II clinical trials for treating pancreatic cancer. Similar mechanisms are employed by other ARBs like irbesartan, valsartan, telmisartan, and olmesartan. Captopril inhibits colorectal liver metastases by reducing tumor viability and hindering metastasis formation. Carvedilol has been found to inhibit breast cancer cell invasion and metastasis by interfering with growth factor receptors and mitochondrial function [22,82,83].

Propranolol, the first effective beta-blocker, is used to treat cardiac illness, hypertension, and conditions like infantile hemangiomas. Cumulative studies have shown that beta-blockers, including propranolol, possess anti-proliferative properties and can inhibit the spread of various cancers, such as ovarian, colorectal, lung, and prostate cancer. Better clinical outcomes in multiple myeloma have also been observed. One hypothesis is that beta-blockers inhibit the sympathetic nervous system in the bone marrow niche, thereby enhancing the clinical outcomes for patients with multiple myeloma, especially those undergoing hematopoietic stem cell transplantation. Clinical trials indicate that propranolol, combined with hematopoietic stem cell transplantation, can be used to treat multiple myeloma despite some adverse events like dizziness, hypotension, and hypokalemia [84,85].

Research suggests that COX-2 inhibitors and beta-blockers together may slow disease progression in some cancer types. COX-2 inhibitors have anti-angiogenic and apoptotic effects, and some cancers, like renal cell carcinoma, release prostaglandins to evade eradication. Preoperative beta-blockers combined with COX-2 inhibitors may enhance immunological response and reduce metastasis. Propranolol and etodolac-treated patients with primary operable breast cancer showed increased tumor-infiltrating β-cells, decreased tumor-infiltrating monocytes, and reduced premetastatic/pro-inflammatory transcription factor activity. Nausea was the most frequent adverse event, with no moderate or severe ones reported [81,86,87].

Preclinical studies have demonstrated that certain antihypertensive drugs augment tumor susceptibility to chemotherapy, decrease cell proliferation, and function as co-adjuvants against chemo-resistant cell lines in different types of cancer. Evidence from a retrospective study suggests that the use of propranolol for a duration of over 1000 days reduces the likelihood of acquiring malignancies in the prostate, colon, stomach, and esophagus. Additional evidence indicates that statins, ACE inhibitors, and ARBs decrease the likelihood of breast cancer recurring. Combined administration of sunitinib with ARBs and ACE inhibitors enhances overall survival, response rate, and progression-free survival and decreases initial treatment relapse in patients with renal cell carcinoma [88,89,90].

Candesartan and irbesartan (ARBs) are inhibitors of angiogenesis and tumor vascularization in colon cancer cell lines. Furthermore, irbesartan inhibits the JUN gene and AP-1 DNA binding in patients with metastatic colon cancer, resulting in a total elimination in radiological testing. The efficacy of ACE inhibitors such as captopril and enalapril in inhibiting the conversion of angiotensin I to angiotensin II has been demonstrated against colon cancer cells both in vitro and in vivo. Enalapril alone decreases the expression of IGF-IR 1 when coupled with 5-fluorouracil, and it enhances radiosensitivity via activating the NF-κB/STAT3 pathway. While enalapril is not now undergoing clinical studies, the existing data indicate its promise as a modified immunotherapy for cancer. The ca channel blocker, nifedipine, solely and selectively suppresses the expression of PDL-1 in colon cancer cell lines [83,91,92].

Eighteen clinical trials have assessed propranolol, a non-selective beta-blocker, alone or with other therapies against various cancers, including pancreatic, neuroblastoma, angiosarcoma, breast, lung, melanoma, and leukemia. Propranolol in combination with etodolac is currently undergoing Phase II and III trials for the treatment of colorectal cancer. In vivo, propranolol reduces the expression of p-AKT/p-ERK/p-MEK and stimulates autologous CD8^+^ T cells, thereby suppressing colorectal cancer. Two Phase I trials are currently being conducted to evaluate the effects of propranolol on ovarian cancer. Its mechanism of action involves activating protective autophagy and apoptosis through the JNK signaling pathway and ROS [93,94].

Two antihypertensive medications are in Phase II trials for prostate cancer: carvedilol and propranolol. Carvedilol is assessed before surgery, while propranolol is examined preoperatively alone or with etodolac. A retrospective study shows that long-term atenolol use significantly reduces prostate cancer risk by over 50% [95,96].

In vitro and in vivo studies show that captopril and candesartan inhibit prostate cancer cells despite not undergoing clinical trials. Captopril increases p53 expression, inducing apoptosis in prostate cancer cells, while candesartan reduces tumor growth and angiogenesis by inhibiting VEGF expression. Hydralazine induces demethylation in prostate cancer cells, re-expressing suppressed genes [97,98].

A Phase II trial is currently underway to evaluate minoxidil, a direct vasodilator antihypertensive medication, as a single treatment for ovarian cancer. By activating a caspase-3 independent cell death pathway, it induces mitochondrial disruption and substantial DNA damage, hence modifying the metabolic and oxidative state of cancer cells [99,100].

The activation of a caspase-3 independent cell death pathway by minoxidil results in mitochondrial disruption, substantial DNA damage, and alterations in the metabolic and oxidative state of cancer cells [101].

Although there are no clinical trials specifically assessing their efficacy, four anti-hypertensive drugs, including captopril, telmisartan, irbesartan, and candesartan, have demonstrated promise in combating liver and kidney cancer in both in vitro and in vivo studies. Telmisartan and irbesartan inhibit the proliferation of liver cancer cells by downregulating the expression of pErbB3 and phosphorylating p38/MAPK, therefore lowering the expression of VCAM-1 downstream [102].

Telmisartan triggers apoptosis in kidney or renal cancer cell lines by increasing the expression of caspase-3 and Bax while decreasing the expression of Bcl-2 (PI3/AKT pathway). Furthermore, captopril suppresses the proliferation of SN12K-1 kidney cancer cells in laboratory and animal models [103].

Tezosentan has been demonstrated to possess the potential to serve as a novel anticancer agent by inhibiting endothelin receptors, which are overexpressed in a variety of cancer cells.

Furthermore, tezosentan has demonstrated promising preclinical results in the inhibition of cancer cell proliferation and the induction of apoptosis, particularly in malignancies with the highest expression of endothelin receptor type A [104,105].

- Antihyperlipidemic

Fenofibrate, initially developed for treating hyperlipidemia, has shown anticancer properties in various human cancer types. Fenofibrate activates AMPK, reduces ATP levels, and increases ROS accumulation, leading to apoptosis and inhibition of metastasis in cancer cells. Fenofibrate also promotes apoptosis and cell cycle arrest by modulating growth factor receptors and NF-κB activity in breast cancer and also inhibiting ERK signaling in lung cancer. Fenofibrate’s anticancer effects are independent of PPARα, making it a potential candidate for repurposing in cancer treatment [106,107].

- Repurposing Ion Channels Modulators for Cancer Treatment

Ion channels play a critical role in specific excitable cells like cardiac myocytes and neurons. The study of ion channels’ roles in the pathophysiology of various diseases has led to the development of drugs targeting these channels. Groundbreaking research highlighting the role of K^+^ channels in mitogenesis and oncogenesis spurred interest in their potential as targets for cancer treatment. Consequently, ion channels are now considered potential therapeutic targets, as they influence cancer cell growth through various mechanisms, including regulating membrane potential and critical survival signaling pathways like Ca^2+^ and Na^+^/K^+^ [108,109,110].

During different stages of the cell cycle, cancer cells experience volume changes controlled primarily by ions such as potassium, calcium, sodium, and chloride. Cancer cells use water flow in restricted areas to propel themselves during migration, with aquaporin channels facilitating water passage between cells. According to the “osmotic engine model”, AQP5 (aquaporin 5) and NHE-1 (Na^+^/H^+^ exchanger-1) are polarized at the cell’s leading edge during migration, allowing water to enter at the front and exit at the rear, resulting in cell displacement [111,112].

With 77 genes encoding K^+^ channels, they are highly diverse, and any member of this large family can be dysregulated in a particular cancer type. Among these, Kv10.1 (EAG1) channels have been extensively studied and are often upregulated in cancer cells. Therefore, specific blockers for these various K^+^ channels are required for optimal function, opening the possibility of tailored medication. Identifying the dysregulated K^+^ channel in a patient allows for treatment with a specific inhibitor of that K^+^ channel family member. For example, Kv10.1 is expressed in about 70% of cancers but is almost absent from non-CNS organs, making it less harmful to target these channels in tumors than in normal tissues [113,114].

Although Kv10.1 is virtually absent in healthy non-CNS tissues, it is significantly upregulated in several cancer cell lines, such as neuroblastoma, cervical carcinoma, melanoma, and breast cancer. An overexpression of Kv11.1 (hERG) is observed in human leukemia cell lines. Kv10.1 channels are notably upregulated in patient tumor tissues, such as soft tissue sarcoma (70%), cervical cancer (100%), gliomas (Kv10.1 and Kv11.1), endometrial cancer (Kv11.1), and chronic lymphocytic leukemia (Kv11.1), as compared to non-cancerous cells. After conducting an extensive analysis of more than 470 clinical tumor samples, it was determined that Kv10.1 was over-expressed in 15 out of 17 different carcinoma types [115,116].

Preclinical research on potassium channel inhibition’s anticancer effects primarily focuses on Kv10.1, Kv10.2 (EAG2), and Kv11.1 channels. Existing drugs, like the antiarrhythmic verapamil, anti-epileptic retigabine, and anti-diabetic sulfonylureas, are known for their inhibitory effects on potassium channels such as KATP, Kv11.1, and KCNQ, respectively.

Sulfonylurea medications, like glipalamide, have shown efficacy against melanoma, lung, stomach, and breast cancers. Verapamil, an antiarrhythmic, is effective against neuroblastoma and prostate cancer. Its combination of inhibitory action against potassium and calcium channels contributes to its anticancer effects. Astemizole, originally developed as an H1-antagonist, now shows potential as an anticancer medication due to its ability to inhibit Kv10.1. Current research suggests that potassium channel blockers could be repurposed as anticancer drugs [117,118].

Calcium channel blockers (CCBs) of the T-type can halt the cell cycle in the G1 or S phases and increase its susceptibility to chemotherapy. Combining CCBs with existing drugs enhances their effectiveness. Additionally, CCBs block p-glycoproteins, which contribute to cancer cells’ multi-drug resistance [102,119,120].

Mibefradil, a T- and L-type Ca^2+^ channel blocker, was initially marketed as an antihypertensive medication but was withdrawn due to interactions with other drugs. Modifications to mibefradil could create more selective T-type Ca^2+^ channel blockers with less CYP450 3A4 inhibition, potentially improving their use in cancer therapy. TAU Therapeutics LLC has repositioned mibefradil for the treatment of high-grade glioma tumors using Interlaced Therapy TM, combining it with temozolomide to enhance the chemotherapy drug’s efficacy in S-phase active cells. This approach is currently undergoing Phase 1b clinical trials to validate its effectiveness [4,121].

#### 2.1.6. Repurposing Antibiotic Medications for Cancer Treatment

Antibiotics are being researched for their potential to target cancer cells in addition to treating bacterial infections. Some antibiotics, including anti-TB medications, tetracyclines, and β-lactam antibiotics, show antitumor activity. Bedaquiline, an antibiotic approved by the FDA for treating pulmonary tuberculosis, has been shown to suppress the development and multiplication of MCF7-derived stem-like cancer cells, as well as reduce mitochondrial oxygen consumption. The promise of bedaquiline as a cancer chemotherapeutic option arises from its ability to target mitochondrial ATP-synthase, which may result in m-ATP depletion and mitochondrial malfunction in cancer cells. However, further research is required to confirm this hypothesis [122,123,124].

Tetracyclines, such as tigecycline and doxycycline, can impede the spread of cancer by specifically targeting the system of oxidative mitochondrial metabolism. By targeting pathways such as AMPK-mediated mTOR, WNT/b-catenin, and PI3K/AKT, they elicit oxidative stress, autophagy, cell cycle arrest, and eventually apoptosis in cancer cells, suppressing cell proliferation, migration, invasion, and angiogenesis [78,125,126].

Clofoctol, a medical agent employed in France and Italy for the treatment of upper respiratory tract infections, has the ability to inhibit protein translation in mammalian cells, therefore indicating its potential utility in the field of cancer therapy. Clofoctol promotes endoplasmic reticulum (ER) stress via activating the unfolded protein response (UPR) pathway, resulting in G1 phase cell cycle arrest in cancer cells. Given that many cancer cells exhibit elevated levels of ER stress, additional augmentation of ER stress may elicit exaggerated stress responses, leading to cell cycle arrest or cell death [127,128].

Anthracyclines (AC), such as doxorubicin, daunorubicin, epirubicin, and idarubicin, intercalate between DNA base pairs, forming a ternary complex with DNA and Topoisomerase II that inhibits DNA and RNA synthesis in actively reproducing cancer cells. Doxorubicin-containing chemotherapy regimens, including FAC (5-fluorouracil, AC), TAC (taxotere, AC), and AC (adriamycin, cyclophosphamide), have been effective in treating breast cancer. Myocet, a non-PEGylated liposomal form of doxorubicin, is licensed for metastatic breast cancer treatment in Europe and Canada when used with cyclophosphamide [129,130,131].

The broad-spectrum tetracycline antibiotic minocycline has demonstrated effectiveness in the treatment of several forms of cancer. Cell cycle arrest and down-regulation of cyclins A, B, and E are mechanisms by which it inhibits the proliferation of ovarian cancer cells. The concurrent administration of minocycline and celecoxib in breast cancer causes a decrease in tumor cell proliferation, microvessel density, and the synthesis of matrix metalloproteinase and VEGF. Autophagy activation is the mechanism by which minocycline suppresses glioma cell proliferation. Ongoing clinical trials are evaluating its capacity to enhance the outcomes of cancer patients by mitigating the adverse effects of chemotherapy [132,133].

The broad-spectrum tetracycline antibiotic tigecycline is effective against multi-resistant bacteria and has anticancer properties, including inhibition of gliomas, myeloid leukemia, non-small cell lung cancer, and retinoblastoma transcriptional corepressor 1 negative breast cancer. Current Phase I trials are investigating the use of tigecycline for the treatment of acute myeloid leukemia [126,134,135].

Fluoroquinolones, including ciprofloxacin, moxifloxacin, levofloxacin, enoxacin, and fleroxacin, disrupt DNA synthesis by inhibiting bacterial gyrase and exhibiting strong anticancer properties. Ciprofloxacin, for example, has been shown to inhibit leukemia, osteoblastoma, osteosarcoma, colon, bladder, and prostate cancers. Enoxacin modulates miRNA production, specifically in cancer, highlighting the potential of fluoroquinolones as anticancer medications [136,137].

#### 2.1.7. Repurposing Anti-Malarial Medications for Cancer Treatment

Chloroquine, artesunate, and mefloquine, originally developed to treat malaria, have shown efficacy against various diseases, including cancer. Chloroquine suppresses autophagy, slows tumor development in glioblastoma xenografts, and enhances the overall outcome when used with chemotherapy drugs. Artesunate induces ROS production and apoptosis in T-cell leukemia and slows tumor growth and angiogenesis in Kaposi’s sarcoma [138].

Mefloquine improves the effectiveness of other therapeutic agents in treating several types of cancer, including breast, leukemia, gastric, cervical, and colon cancers, by decreasing the expression of P-gp, blocking cell division, and disrupting lysosomes through the production of ROS [139,140].

#### 2.1.8. Repurposing Antipsychotic Medications for Cancer Treatment

Antipsychotic medications have been shown to possess anticancer properties, affecting biochemical pathways that result in tumor cell death. Haloperidol and penfluridol, hydroxypiperidine-based antipsychotics, inhibit DRD2 activity, promoting ER stress and suppressing pancreatic cancer cell proliferation with minimal toxicity. Penfluridol stimulates autophagy and death in malignant pancreatic cells and has synergistic effects when combined with gemcitabine. Penfluridol specifically affects the JAK2 binding site in PRLR, therefore blocking the JAK2–STAT3 and ERK/AKT signaling pathways. This action effectively inhibits the proliferation of pancreatic cancer cells [141,142,143].

#### 2.1.9. Repurposing NSAID Medications for Cancer Treatment

Chronic inflammation is linked to cancer risk, with inflammatory mediators like growth factors, chemokines, and cytokines facilitating cancer cell survival and proliferation. NSAIDs, such as mesalazine, sulindac, piroxicam, and ibuprofen, have shown potential in reducing tumor growth and cancer recurrence. Diclofenac has been found to slow pancreatic tumor growth, while the selective COX-2 inhibitor, celecoxib, inhibits breast cancer cell proliferation and tumor formation. The correlation between NSAID usage and slower cancer development suggests potential repurposing opportunities [144,145].

#### 2.1.10. Repurposing Disease-Modifying Antirheumatic Drug (DMARD) for Cancer Treatment

Auranofin, a disease-modifying antirheumatic drug (DMARD), was once used to treat rheumatoid arthritis by suppressing inflammation and stimulating cell-mediated immunity. However, it was found to be less effective than other DMARDs, such as methotrexate, and its use declined due to its adverse effects, including gastrointestinal complaints, skin irritations, and oral ulcerations. However, it has the potential for novel applications in treating certain malignancies. Auranofin is effective against cancer cells through two mechanisms: inhibiting mammalian TrxR (mTrxR) and targeting the UPS system (Understanding the Unstable Cells) system. These mechanisms induce apoptosis, indicating that auranofin may no longer be the preferred medication for rheumatoid arthritis [146,147]. Not only the gold complex, auranofin, which showed promising anticancer effects, but other metal complexes with clinically approved drugs having various therapeutic applications also led to the repositioning of such complexes for cancer therapy. Metformin-decavanadate, vanadium-bisphosphonates, vanadyl(IV) complexes with non-steroidal anti-inflammatory drugs, and cetirizine and imidazole-based oxidovanadium(IV) complexes are examples [147,148].

#### 2.1.11. Repurposing Anti-Epileptic Medications for Cancer Treatment

Oxcarbazepine is a commonly used pharmaceutical agent specifically designed to treat epilepsy. Numerous preclinical studies have provided evidence of anticancer capabilities. One study has shown that oxcarbazepine results in cell cycle arrest, specifically during mitosis. Additional studies suggest that it works as an inhibitor of HDAC, therefore suppressing the subsequent PI3K-Akt-mTOR pathway. The ultimate result of this inhibition is the prevention of cell growth and migration. Ultimately, this suppression hinders the manifestation of mTOR and the proteins Bax and Bcl-2, facilitating the expression of caspase-3 and caspase9, leading to apoptosis and autophagy [149,150].

The compound lacosamide belongs to the third generation of anti-epileptic drugs, which improve the delayed deactivation of voltage-gated Na^+^ channels. It is used as an adjunctive treatment as it has the capacity to reduce seizure frequency without substantially affecting mood [150]. Lacosamide was found to also inhibit HDAC during processing. This activity may indicate the need to undertake an investigation into its anticancer properties. Moreover, this process has been suggested as a possible rationale for the inhibition of cell cycle migration in glioma cells, which may be triggered by the increase in miR-195-5p levels [151]. The same research proposed that lacosamide might dampen cellular proliferation, impede cell migration and invasions, and stimulate apoptotic processes by altering the production of other microRNAs, such as miR-107. A study team presented evidence indicating that the phosphorylation of collapsin-response-mediator-protein (CRMP2) is a strong indicator of glioblastoma cell proliferation [151,152].

In order to examine the impact of CRMP2 phosphorylation at S522 on tumor growth, researchers utilized the CRMP2 phosphorylation inhibitor (S)-lacosamide. Inhibiting CRMP2 phosphorylation with (S)-lacosamide was shown to decrease the proliferation of glioblastoma cells in all glioblastoma cell lines. Furthermore, they provided evidence that (S)-lacosamide suppresses the growth of glioblastoma in live animal models [153,154].

Lamotrigine is another anti-epileptic medication that inhibits salt transport. Aside from primarily blocking voltage-gated Na^+^ channels, it also inhibits N-, L-, and P-type calcium channels and, to a lesser degree, 5-HT3 receptors. Through the reduction in glutamate synthesis, these effects enhance the stability of neuronal membranes. Similar to lacosamide, lamotrigine is a potential adjunctive drug for patients diagnosed with brain tumors. Published research suggests that pairing it with valproic acid can enhance the treatment of refractory epilepsies by leveraging synergy [155,156].

#### 2.1.12. Repurposing Anesthetic Medications for Cancer Treatment

Ketamine is primarily used in the induction and maintenance of anesthesia due to its antagonistic action on the N-methyl-D-aspartate (NMDA) receptor. It produces a dissociative anesthesia described as a trance-like state, and it can also cause amnesia and alleviate pain. Therefore, ketamine is recommended for cancer patients in order to minimize pain. Furthermore, initial investigations have shown that the introduction of ketamine to specific cells leads to the cessation of the cell cycle and the suppression of cell division, therefore suggesting a possible future use of ketamine in the prevention of cancer advancement. Zhou et al. [157] showed that the administration of ketamine promotes apoptosis in lung cancer cells by increasing the expression of CD69, a marker associated with the activation of leukocytes and natural killer (NK) cells. Ketamine exerts its anticancer properties in ovarian cancer by specifically reducing the expression of RNA PVT1. Moreover, ketamine therapy decreased the interaction between PVT1 and histone methyltransferase enhancer of zeste homolog 2. The aforementioned relationship leads to the increase in the expression of P57, a cell cycle inhibitor that subsequently hinders the advancement of the cell cycle in malignant cells. In breast cancer cells and hepatocellular carcinomas, the administration of ketamine can inhibit cell growth and trigger ferroptosis, as well as apoptosis, by specifically inhibiting glutathione peroxidase 4 [52].

Propofol is a rapid, effective intravenous anesthetic with analgesic, hypnotic, and sedative properties. It elicits the central inhibitory neurotransmitter response by activating central GABA receptors, similar to barbiturates and benzodiazepines. Propofol has been found to have anti-inflammatory properties and may enhance the immunological response in older individuals. Studies show that propofol can reduce the cytotoxic activity of NK cells in individuals with gastrointestinal conditions while increasing their efficacy in patients with propofol anesthesia. Propofol has also been studied for its anti-proliferation effects in various cancer cells, suppressing cell replication and stimulating caspase and MAPK pathways. It can induce apoptosis in oral squamous cell cancer resistant to 5-fluorouracil and reverse the resistance of OSCC to 5-fluorouniprolactone [158].

### 2.2. Repurposing with Type-Specific Anticancer Activity

#### 2.2.1. Repurposing Medications for Prostate Cancer (PC) Treatment

- Anti-Dyslipidemic Drugs

Prostate cancer (PC) severity is linked to circulating androgen levels, primarily derived from cholesterol. PC cells rely on endogenous cholesterol production, making patients with high serum cholesterol levels more susceptible to PC. Statins, which inhibit HMG-CoA reductase, block cholesterol synthesis and androgen production, enhancing prognosis, reducing tumor volume, and decreasing PSA levels. Statins like atorvastatin have shown potential in PC treatment, either as monotherapy or in combination [159,160].

- Antiarrhythmic Drugs

The sodium–potassium pump is inhibited by cardiac glycosides, including digoxin and ouabain, resulting in intracellular calcium buildup and death of PC cells. Furthermore, these medications stimulate cell cycle arrest and autophagy. Digoxin suppresses the activity of topoisomerase II and lowers the levels of PSA in the blood, while ouabain increases the susceptibility of PC cells to apoptosis by blocking the production of survivin and STAT3. Ouabain also demonstrates anticancer cytotoxicity in castration-resistant PC [160,161].

**Table 1:** Repurposed drugs for cancer treatment with their chemical structure, new indication, old and new targets, and development status.

Pharmacological Class	Drug Name	Chemical Structure	New Therapeutic Indication	New Target	Original Target	Development Status	References
*Anti-Platelet*	Aspirin	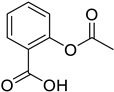	Gastric, esophageal, colorectal, pancreatic, ovarian, endometrial, breast, and prostate cancers	PIK3CA, mTORC1 and AMPK	COXs	Phase II (NCT00468910) and III (NCT02301286) clinical trials, meta-analysis	[32,33,34,35,36,37,38,39,40]
*Anti-Diabetic*	Metformin (biguanides)	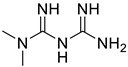	Colorectal, breast, pancreatic, prostate, lung, and cervical malignancies	Cell cycle/pSTAT3, S6 kinase, and mTOR/AMPK/	Mitochondrial respiration	Phase II (NCT05929495) and III (NCT03685409) clinical trials	[41,42,43,44,45,46,47,48,49,59,60,61,162,163,164,165,166,167,168,169,170,171]
	Pioglitazone (TZDs)	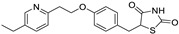	Breast, prostate, and colon cancer	PPAR γ	PPAR γ	Phase II trials (NCT00099021)	[55,56,57,58,59,60,61]
	Desmopressin	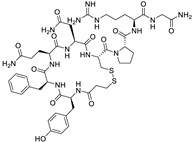	Colon cancer	COX-2 and CD1	AVPR2	Preclinical	[30,52,53,54]
*Anti-Helminthic*	Flubendazole (benzimidazole)	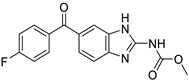	Neuroblastoma, multiple myeloma, leukemia, lung, liver, colorectal, and breast cancer	Apoptosis (caspase 3 and 7)	Tubulin polymerization	Preclinical	[64,65,66,67,68]
	Parbendazole	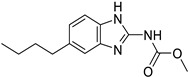	Pancreatic cancer	Apoptosis, cell cycle, and DNA damage	Tubulin polymerization	Preclinical	[52]
	Mebendazole (MZ)	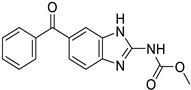	Glioblastoma, melanoma, prostate, breast, brain, ovarian, colon, lung, colorectal and endocrine cancers	Cell cycle, apoptosis (caspase-3 pathway), ABL and BRAF	Tubulin polymerization	Preclinical	[62,63,64,69,70,71,72,172,173]
	Niclosamide	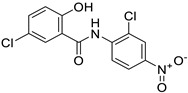	Colon, prostate, liver, ovarian, and breast cancers	Wnt/β-catenin, NF-KB, mTOR, and JAK/STAT3 pathways	Uncoupling of oxidative phosphorylation	Preclinical	[66,73,74,162,163,164]
	Clioquinol	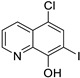	Leukemia and malignant myeloma	HDAC	DNA replication	Preclinical	[75,76]
*Anti-Viral*	Ritonavir	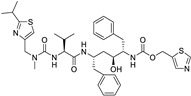	Ovarian, pancreatic, and breast cancer, lymphocytic leukemia	Apoptosis	Protease inhibitors target HIV	Preclinical	[78,79,80]
	Ribavirin	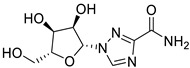	Acute myeloid leukemia (AML)	Induces VEGF mRNA translation	RNA replicating	Phase II clinical trial (NCT00559091)	[81]
	Cidofovir	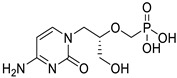	Glioblastomas	Apoptosis	Viral DNA polymerase	Preclinical	[81]
*Cardiovascular* *Anti-Hypertension Medication*							
*Angiotensin Receptor Blocker*	Losartan	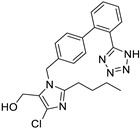	Pancreatic cancer	Depleting the matrix and reducing collagen I levels	Angiotensin receptor	Phase II clinical trials (NCT01821729)	[22,82,83,89]
	Candesartan	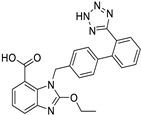	Colon cancer, prostate cancer, liver and kidney cancer	VEGF expression	Angiotensin receptor	Preclinical	[83,91,92,97,98,102]
	Irbesartan	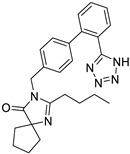	Colon cancer, liver and kidney cancer	AP-1 DNA binding, pErbB3, and p38/MAPK	Angiotensin receptor	Preclinical	[22,82,83,91,92,102]
	Telmisartan	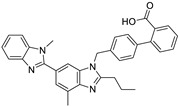	Colon cancer, liver and kidney cancer	pErbB3, p38/MAPK caspase-3, Bcl-2, PI3/AKT pathway	Angiotensin receptor	Preclinical	[22,82,83,102,103]
*ACE Inhibitors*	Captopril	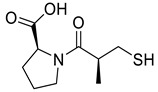	Colorectal liver metastases, prostate cancer, liver and kidney cancer	p53 expression	ACE	Preclinical	[22,82,83,91,92,97,98,102,103]
	Enalapril	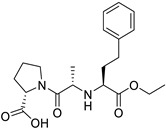	Colorectal cancer (CRC)	IGF-IR 1	ACE	Preclinical	[83,91,92]
*Beta-Blockers*	Carvedilol	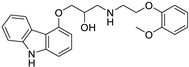	Breast cancer	Growth factor receptors and mitochondrial function	Beta receptors	Phase II trials (NCT02177175)	[22,82,83,95,96]
	Propranolol	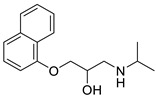	Ovarian, colorectal, lung, prostate, breast cancer, multiple myeloma, pancreatic, neuroblastoma, angiosarcoma, melanoma, and leukemia	p-AKT/p-ERK/p-MEK and CD8^+^ T cells JNK signaling pathway and ROS.	Beta receptors	Phase I trials (NCT03633747) Phase II trials (NCT02596867)	[81,84,85,86,87,88,89,90,95,96]
*Direct Vasodilator*	Minoxidil	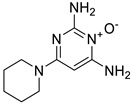	Ovarian cancer	Caspase-3	ATP-sensitive potassium channels	Phase II trials (NCT05272462)	[99,100,101]
	Hydralazine	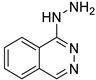	Prostate cancer	Induces demethylation, re-expressing suppressed genes	Direct vasodilator	Preclinical	[97,98]
	Tezosentan	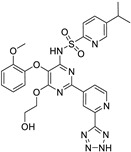	Various cancer types, especially with high expression of endothelin receptor type A	Endothelin receptor A	Endothelin receptor A/B	Preclinical	[104,105]
*Cardiovascular* *Antihyperlipidemic*	Fenofibrate	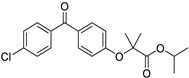	Breast cancer, lung cancer	AMPK, NF-κB, and ERK signaling	PPARα	Preclinical	[106,107]
*Ion Channels modulators*							
*Potassium K^+^ Channel Inhibitors*	Glipalamide	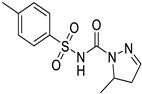	Melanoma, lung, stomach, and breast cancers	Kv10.1, Kv10.2 (EAG2), and Kv11.1 channels	K channel (SUR).	Preclinical	[117,118]
	Verapamil	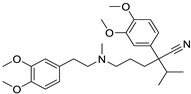	Neuroblastoma and prostate cancer	K and Ca channels	T- and L-type Ca^2+^ channel antagonist	Preclinical	[117,118]
	Astemizole	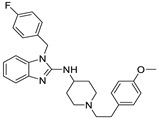	Various cancer cell lines	Kv10.1	H1-antagonist	Preclinical	[117,118]
*Calcium (Cav) Channel Blockers*	Mibefradil	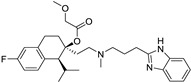	High-grade glioma tumors	T-type Ca^2+^ channel	T and L-type Ca^2+^ channel	Phase I trials (NCT01480050)	[4,121]
	Nifedipine	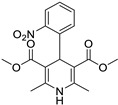	Colon cancer	PDL-1	Calcium channel	Preclinical	[83,91,92]
*Antibiotic*	Bedaquiline	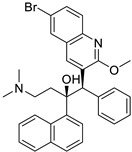	Breast	Mitochondrial ATP-synthase	ATP synthase	Preclinical	[122,123,124]
	Doxycycline (Tetracycline)	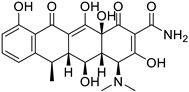	Various cancer cell lines	AMPK-mediated mTOR, WNT/b-catenin, and PI3K/AKT	30S ribosomal subunit	Preclinical	[78,125,126,132,133,134,135,174,175,176]
	Clofoctol	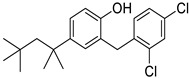	Various cancer cell lines	UPR pathway	Bacterial protein synthesis	Preclinical	[127,128]
	Doxorubicin (Anthracyclines)	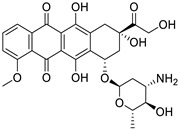	Breast cancer	DNA intercalator	DNA intercalator	Approved	[129,130,131]
	Minocycline (Tetracycline)	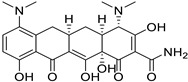	Ovarian, breast cancer, glioblastoma	Cell cycle arrest, cyclins A, B, and E	Inhibit the 30S ribosomal subunit	Phase II trials (NCT01580969)	[132]
	Tigecycline (Tetracycline)	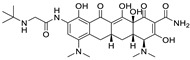	Gliomas, myeloid leukemia, non-small cell lung cancer	Cell cycle arrest	Inhibit the 30S ribosomal subunit	Phase I trials (NCT01332786)	[125,126,134,135,177,178]
	Ciprofloxacin (Fluoroquinolones)	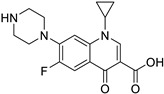	Leukemia, osteoblastoma, osteosarcoma, colon, bladder, and prostate cancers	miRNA production	Inhibit bacterial gyrase	Preclinical	[136,137]
*Anti-Malarial*	Chloroquine	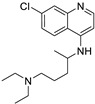	Glioblastoma	Autophagy	Inhibits heme polymerase	Preclinical	[62,63,138,139,140]
	Artesunate	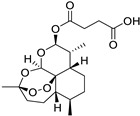	Leukemia, Kaposi’s sarcoma	ROS production and apoptosis	Free radicals generation	Preclinical	[138]
	Mefloquine	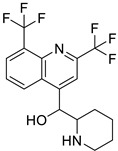	Breast, leukemia, gastric, cervical, and colon cancers	P-gp expression, production of ROS	Inhibits 80S ribosome	Preclinical	[138,139,140]
*Antipsychotic*	Haloperidol	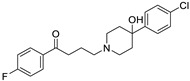	Pancreatic cancer	DRD2	DRD2	Preclinical	[141,142,143]
	Penfluridol	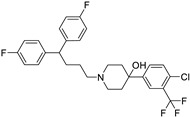	Pancreatic cancer	DRD2, autophagy, JAK2–STAT3 and ERK/AKT signaling pathways	DRD2	Preclinical	[141,142,143]
*Nonsteroidal Anti-Inflammatory Drug (NSAID)*	Diclofenac	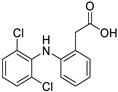	Pancreatic cancer	Wnt/β-catenin signaling pathway	COXs	Preclinical	[144,145,179]
	Celecoxib (selective COX-2 inhibitor)	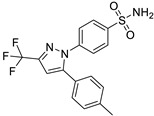	Breast cancer	Wnt/β-catenin signaling pathway	COX-2	Phase II trials (NCT01695226)	[132,133]
*Disease-Modifying Antirheumatic Drug (DMARD)*	Auranofin	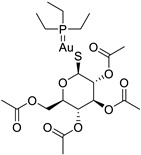	Various cancer types	TrxR, UPS system	Redox enzymes	Phase I (NCT01737502) and Phase II (NCT01419691) trials	[146,147,148]
*Anti-Epileptic*	Oxcarbazepine	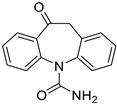	Various cancer types	Cell cycle arrest, HDAC, PI3K-Akt-mTOR pathway	Na channel inhibitor	Preclinical	[149,150]
	Lacosamide	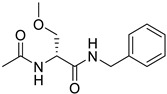	Glioblastoma	CRMP2	Na channel	Preclinical	[151,152,153,154,155,156]
	Lamotrigine	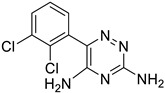	Brain tumors	N-, L-, and P-type Ca channels, 5-HT3 receptors	Na^+^ channels	Preclinical	[155,156]
*Anesthetic Medications*	Ketamine	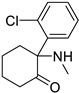	Lung cancer, ovarian cancer, breast cancer, hepatocellular carcinomas	CD69, P57, glutathione peroxidase 4	NMDA receptor	Preclinical	[52]
	Propofol	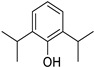	Squamous cell carcinoma	Caspase and MAPK pathways	GABA receptors	Preclinical	[157,158]

- Anti-Inflammatory Drugs

Indomethacin, a non-selective COX inhibitor, inhibits angiogenesis and cancer cell growth through various pathways. Diclofenac inhibits COX, prostaglandin synthesis, and MYC gene family expression, disrupting glucose metabolism in PC cells. Aspirin inhibits COX, prostaglandins, and NF-κB, making it a potential candidate for PC treatment, especially when combined with statins [179].

- Antidepressant Drugs

Sertraline, a selective serotonin reuptake inhibitor, reduces PC cell proliferation and induces apoptosis through mitochondrial dysfunction and ROS production. Sertraline also inhibits tumorigenesis, angiogenesis, and metastasis, disrupting PC stem cells’ viability by modulating iron homeostasis. Stress mitigation is crucial for successful cancer treatment, and sertraline has shown potential for reducing PC cell viability, inhibiting cell proliferation, and enhancing autophagy [180].

- Beta-Blockers

Beta-blockers are cardioprotective medications with potential anticancer properties. They inhibit β-adrenergic receptors involved in carcinogenesis, angiogenesis, and tumor metastasis. Beta-blockers have shown promising results in reducing cancer cell proliferation, migration, and tumor growth, particularly in neuroendocrine prostate cancer (NEPCA). Propranolol disrupts the CREB1-EZH2-TSP1 pathway, down-regulating NE markers and promoting xenograft development from NEPCA cells [181].

- Miscellaneous

Heparin, an anticoagulant, has shown potential as an anti-inflammatory and antimetastatic agent in cancer treatment. It inhibits cytokine, adhesion molecule, and angiogenic factor expression, disrupting the tumor-mediated coagulation cascade. Low molecular weight heparin (LMWH) exhibits antiangiogenic and antimetastatic effects, significantly reducing metastases in a murine CRPCA model [182].

The bisphosphonate medication zoledronic acid blocks bone resorption by suppressing osteoclast activity. Therefore, it is employed in the management of osteoporosis. The therapeutic application of this repurposed medication in the management of prostate cancer has received approval. Corey and colleagues showed that the anti-PC properties of zoledronic acid in vitro cause G1 arrest, deceleration of cell proliferation, and death, and in vivo by reducing the ability to metastasize. Zinc azithromycin effectively decreases skeletal-related problems in PC patients with bone metastases, while it suppresses the growth of osteoblastic and osteolytic metastases in PC. Celecoxib, a COX-2 inhibitor, in combination with zametaxel did not improve survival rates in advanced PC patients. However, docetaxel and zaetaxel significantly improved prognosis in PC patients. Mifepristone, a potent anti-progesterone, has shown anticancer properties in androgen-sensitive and androgen-insensitive PC. It inhibits tumor proliferation by suppressing over-expressed cell surface receptors in CRPC cells. Rapamycin, an anti-restenosis medication and immunosuppressant for kidney transplantation, has been repurposed for cancer treatment by binding to the FRB domain of mTOR, inhibiting mTORC1 [183,184].

#### 2.2.2. Repurposed Medications in the Treatment of Gastric Cancer (GC)

- Antidepressants

Fluoxetine is a selective serotonin reuptake inhibitor (SSRI) used to treat depression, anxiety, and post-traumatic stress disorder. It has shown anticancer potential in cancer cells, causing apoptosis and cell cycle disruption. Fluoxetine suppresses cell proliferation in AGS GC cells and induces apoptosis, which is partially suppressed by the inhibition of the endoplasmic reticulum stress marker (CHOP). It also enhances apoptosis and autophagosome formation in AGS cells.

Sertraline, an SSRI, has shown antitumor activity in lung, prostate, breast, melanoma, and acute myeloid leukemia. It induces apoptosis and cell cycle arrest in resistant GC cells, leading to growth advantage, resistance to therapy, angiogenesis, and metastatic potential. Sertraline and its derivatives can be repurposed as chemosensitizers for drug-resistant GC.

Paroxetine, an SSRI, also has anticancer properties by inducing apoptosis and suppressing cancer cell proliferation in the colon, hepatocellular carcinoma, osteosarcoma, and lymphoma. Its cytotoxicity in AGS cells is facilitated by enhanced DNA damage and reduced DNA repair. However, it is incapable of inducing apoptosis or DNA damage in MKN-45 cells. Combining paroxetine with chemotherapy or anticancer agents can enhance GC treatment [185].

- Anti-epileptic

Valproic acid (VPA) is a fatty acid with antiseizure properties and has been shown to possess anticancer properties in various cancers. VPA induces apoptosis and suppresses cell proliferation in AGS GC cells, inhibiting the HDAC1/PTEN/Akt signaling pathway, which influences cell proliferation, differentiation, and immunity. It also enhances Beclin1 expression, a tumor suppressor, and inhibits Bcl-2 expression. However, high HDAC1 and reduced PTEN expression in MKN-74 GC cells render them resistant to VPA treatment. In vivo, VPA inhibits GC cell growth through apoptosis and autophagy and increases the expression of survivin, a protein essential for cell division. In a separate study, VPA exhibited an elevated apoptotic index and decreased tumor volume. However, a randomized Phase II trial showed that VPA does not provide a survival advantage compared to paclitaxel alone in second- or third-line therapy for advanced GC [185,186].

- Antipsychotic

Thioridazine and risperidone are antipsychotic compounds that are employed in the treatment of acute mania, bipolar disorder, and schizophrenia in the psychiatric field. In glioblastoma, lung, and colon malignancies, thioridazine demonstrates antitumor properties. Thioridazine induces apoptosis in a caspase-dependent manner and inhibits colony formation capacity and nuclear fragmentation in NCI-N87 and AGS GC cells, thereby exerting cytotoxic effects in a fashion that reduces the quantity of caspase-9, caspase-8, and caspase-3 precursors. This medication has the capacity to inhibit the growth of tumors in vivo. Risperidone prevents the proliferation of KATO-III GC cells by increasing the level of ROS and inducing apoptosis.

Additionally, this medication inhibits the growth of tumors in vivo. In a population-based cohort study, the anticancer effects of risperidone were demonstrated. Patients who used risperidone had a lower risk of GC compared to those who did not [187,188].

- Agents that chelate iron

Deferasirox, a tridentate iron chelator, has been shown to have antitumor activity in various malignancies, including pancreatic and breast cancers, mantle cell lymphoma, and lymphoma. It suppresses the proliferation of AGS, MKN-28, SNU-484, and SNU-638 GC cells, induces apoptosis, and upregulates N-myc downstream-regulated gene 1, inhibiting metastasis. It also downregulates phospho-mTOR and c-myc expression, suggesting that deferasirox may enhance the antitumor effect of cisplatin in GC [185,189].

#### 2.2.3. Repurposed Medications in the Treatment of Blood Malignancies

Thalidomide was developed in the 1950s, originally as a sedative, and was used to alleviate morning sickness in pregnant women. Following its introduction, thalidomide was withdrawn from the market because of its capacity to cause serious congenital abnormalities. An estimated minimum of 10,000 newborns were born with limb and other body extremity abnormalities in more than 46 countries throughout this timeframe. Over the following decades, scientists persistently explored the medicinal applications of thalidomide [190,191].

Judah Folkman’s laboratory provided evidence that thalidomide significantly decreased the formation of new blood vessels. After careful evaluation in patients with refractory multiple myeloma, the medication demonstrated the ability to produce long-lasting effects by blocking TNF-α. Thalidomide was granted FDA approval in 2006 as a therapeutic option for multiple myeloma when administered in combination with dexamethasone [192,193].

It is rather typical for cancer treatment drugs to be repurposed within the same disease subtype for which they were originally indicated. For example, imatinib mesylate was originally approved for the treatment of chronic myelogenous leukemia. Nevertheless, imatinib displayed cross-reactivity with and the ability to suppress KIT kinase, since it acts as an inhibitor of the ABL kinase. The identification of mutations that activate the KIT tyrosine kinase as a genetic factor contributing to gastrointestinal stromal tumors (GISTs) suggests that imatinib should be evaluated for its effectiveness in treating GISTs. The notion of the possible indication was examined by preclinical analyses, which confirmed that imatinib indeed caused cell death in GIST cells. This phenomenon was theoretically linked to the suppression of KIT kinase. The therapeutic use of imatinib for GISTs was approved based on these studies [193]. The current indication for imatinib is the treatment of certain blood malignancies, such as acute lymphoblastic leukemia, chronic eosinophilic leukemia, and myelodysplastic/myeloproliferative neoplasms. Furthermore, it has received approval for the management of systemic macrocytosis and dermatofibrosarcoma due to an allergic response [194,195].

Dasatinib, originally approved for chronic myeloid leukemia, was subsequently adapted for the treatment of individuals with Philadelphia chromosome-positive acute lymphoblastic leukemia who had shown resistance or intolerance to previous pharmaceutical treatments, akin to imatinib. Dasatinib is an orally administered inhibitor of tyrosine kinase that is actively being studied in clinical studies for the therapy of glioblastoma. Furthermore, it has been used for the therapy of brain metastases caused by breast cancer using network-based techniques, which utilize the reconstruction of disease-specific networks to pinpoint important targets [196,197].

#### 2.2.4. Repurposed Medications in the Treatment of Breast Cancer

Fluorouracil (5-FU) is a heterocyclic aromatic molecule that has a ribinoid structure similar to the pyridine ring present in DNA and RNA. More precisely, the uracil homolog substitutes hydrogen with fluorine at position C-5. Therapeutic use of 5-FU for cerebral palsy patients has been authorized by the US FDA. Basal cell carcinoma is commonly treated with 5-FU.

There are several recognized methods by which it exerts its anticancer effects, including the stimulation of apoptosis and the suppression of cancer growth (e.g., the Ras/ERK pathway). The compound 5-FU exhibited apoptotic effects and reduced the expression of the H-ras gene in in vitro studies using the MDA-MB-231 and Tumor2 cell lines [198].

Moreover, the Ras/ERK pathway is suppressed from eliciting antiproliferative actions. Furthermore, 5-FU reduces the expression of the Rho-A gene, therefore reducing apoptosis and metastasis. 5-FU markedly reduced the expression of Rac1 protein in Tumor2 and MDA-MB-231 cells, which plays a crucial role in cell movement, invasion, and resistance to cell death. 5-FU downregulated the expression of the p53 gene in Tumor2. The activation of NF-κB is commonly detected in breast cancer cells. An investigation found that the proliferation and survival of cancer cells, as well as the expression of the NF-κB gene, were decreased following treatment with 5-FU. A research investigation found that 5-FU decreased protein expression in the tumor2 cell line compared to its equivalents. Multiple results corroborate the notion that NF-κB activation decreases medication resistance to 5-FU in human cancer cell lines. This confers a clear superiority over the presently accessible medications, which are at the pinnacle of drug resistance. Additional studies provide further evidence supporting previous studies, demonstrating that the therapeutic effectiveness of 5-FU has been enhanced as a result of decreased NF-κB expression. The combination of 5-FU with other treatments enhances the probability of a significant response in the setting of drug-resistant cancers [199].

Nitroxoline (NTX) is an antibiotic that is extensively used in Asian, European, and African countries for the effective treatment of urinary tract infections. It is chemically known as 5-nitro-8-hydroxy-quinoline. Nitroxoline is currently experiencing significant popularity due to its potent anticancer properties. The anti-angiogenic activity of nitroxoline was the first anticancer activity to be documented in human bladder cancer. NTX exhibited prospective antineoplastic activity against a variety of malignancies, including lymphoma, glioma, leukemia, breast, bladder, pancreatic, and ovarian cancers [199]. This is particularly noteworthy. NTX has been reported to decrease the volume of tumors in the BC xenografts model by 60% and to inhibit the development of bladder cancer in the orthotopic mouse model. In the MMTV-PyMT and LPB fibrosarcoma breast cancer mouse models, nitroxoline significantly inhibited tumor development, angiogenesis, and metastasis in vivo. The cathepsin B is inhibited by NTX, which is elevated in BC patients. The inhibition of cathepsin B results in a decrease in ECM degradation and a subsequent invasion in 2D and 3D tumor models [200].

NTX significantly reduces tumor growth and metastasis by cooperating with MetAP2, SIRT1, and Cat B in vivo, thereby inhibiting the development of endothelial cell tubes. A study found that NTX eliminates cancer hallmarks such as invasion, angiogenesis, and metastasis in in vivo models, irrespective of the route of administration. It is a superior drug against breast cancer compared to current regimens due to its exceptional pharmacokinetic profile and lack of systemic toxicities [157].

#### 2.2.5. Repurposed Medications in the Treatment of Colon Cancer

- Antihypertensive and antiarrhythmic medications

The drugs ACEIs and ARBs are commonly used and provide significant therapeutic benefits for patients undergoing treatment for many diseases, including hypertension and heart failure. An in vivo investigation revealed that both ACEIs and ARBs effectively suppressed colitis-induced colorectal cancer (CRC) in obese mice by lowering chronic inflammation and oxidative stress. In an independent trial carried out by Kedika et al., individuals who had one or more adenomatous polyps confirmed by histological analysis on a first colonoscopy and were given lisinopril, an antineoplastic chemotherapeutic agent, experienced a 41% reduction in the probability of acquiring similar polyps during the following 3–5 years. Furthermore, studies showed that the use of ACEI/ARB and beta-blockers together resulted in higher survival rates, reduced hospitalization, and slowed tumor progression in advanced colorectal cancer [201,202,203].

Beta-blockers are pharmacological agents classified as class II antiarrhythmic drugs, mainly indicated for the treatment of cardiovascular disorders and several other syndromes. An investigation showed that nebivolol specifically suppresses mitochondrial respiration in an HCT-116 colon cancer cell line. This was accomplished by impeding the function of complex I in the respiratory chain and inhibiting the proliferation of colon cancer cells. Such evidence implies that this medication has the capacity to be repurposed for the treatment of colon cancer [31,201,204].

- Anti-Helminthic

For a range of CRC cell lines, including HCT-116, RKO, HT-29, HT-8, and SW626, mebendazole demonstrates cytotoxic action. Nygren and Larsson observed that mebendazole successfully caused partial regression of metastatic lesions in a patient with refractory metastatic CRC. A separate study found that the combination of mebendazole and sulindac (an NSAID) reduced the quantity and size of intestinal microadenomas in mice with a constitutional mutation in the Adenomatous polyposis coli (APC) gene by blocking the MYC and COX-2 pathways, angiogenesis, and the release of pro-tumorigenic cytokines [62,70,172].

A salicylamide derivative, niclosamide, modulates several signaling pathways and dissociates oxidative phosphorylation. In both laboratory and animal studies, niclosamide suppressed the Wnt/β-catenin cascade, which is abnormally active in 80% of sporadic CRC. Possibly through the stimulation of autophagy, this downregulation led to reduced proliferation in several human CRC cell lines, including HCT-116, Caco2, and HT-29. Moreover, a recent investigation demonstrated that the combination of niclosamide and metformin can effectively decrease Wnt and YAP, thereby cooperatively inhibiting APC-mutant CRC [162,163,164].

Sirolimus, often known as rapamycin, is an FDA-approved inhibitor of the mTOR pathway employed for the prevention of rejection following renal transplantation. The synergistic inhibition of colon tumor growth by a combination of sirolimus and metformin has been demonstrated by Mussin et al. in both in vitro and in vivo settings. In their study, he and his colleagues provided evidence that mTOR inhibitors trigger death in colon cancer cells by promoting the dephosphorylation of 4EBP1 through CHOP-dependent DR5. This process ultimately results in a decrease in tumor growth, angiogenesis, and invasion. It has been demonstrated that sirolimus suppresses mTOR activity, which in turn decreases the migration and invasion of CRC cells, therefore inhibiting the EMT induced by FBXW7 deletion [165,166,167].

#### 2.2.6. Repurposed Medications in the Treatment of Hepatocellular Carcinoma (HCC)

Pimozide, an antipsychotic medication, can potentially suppress the growth of hepatocellular carcinoma (HCC) cell lines in laboratory settings by triggering apoptosis during the G0/G1 phase. Moreover, pimozide exhibited an inhibitory effect on HCC stem-like cells, specifically targeting the CD133-positive cell population. The luciferase assay activity of pimozide specifically targeted the expression of STAT3, leading to a decrease in the transcription levels of downstream oncogenes involved in STAT3 signaling activities. Further validation of the anticancer effect of pimozide was conducted in vivo using nude rodents [142,205,206].

Amiodarone, a class III antiarrhythmic drug and aggressive mTOR inhibitor, was shown to decrease liver tumor development by promoting autophagy activity in both the mouse xenograft model and the rat orthotropic model. Moreover, a comprehensive examination of 32,625 case-control participants from Taiwan’s National Health Insurance program revealed that consistent long-term use of amiodarone significantly decreases the incidence of HCC. Amiodarone, a repurposed medication, has anticancer properties via stimulating autophagy activity, which can inhibit the development of liver tumors and prevent the onset of HCC [207,208].

Lanatoside C is an endogenous compound derived from digitalis lanata, known for its antiarrhythmic properties. The capacity of lanatoside C to modulate HCC both in laboratory settings and in living organisms was shown by incorporating a systems biology repositioning strategy, leading to a significant decrease in tumor proliferation. Molecular mechanistic analysis revealed that lanatoside C can cause cell death by promoting the depletion of mitochondrial membrane potential. Thus, the activation of apoptotic markers ensues. Through the suppression of Thr505 phosphorylation for protein kinase delta (PKCd), lanatoside C reversed lanatoside Ce-induced mitochondrial membrane potential loss and death, validating its molecular mechanism, which involves the AKT/mTOR pathway through control of PKCd activation [207,209].

An FDA-approved medication for pneumocystis pneumonia, atovaquone, significantly suppressed the growth of HCC by arresting the S phase of the cell cycle and enhancing both intrinsic and extrinsic apoptotic pathways, which were linked to the elevated expression of p53 and p21. Through the induction of double-stranded DNA breaks, atovaquone has been demonstrated to suppress hepatoma cell growth by generating prolonged activation of the ataxia telangiectasia mutant and its downstream components, including cell cycle checkpoint kinase-2 and H2AX. Furthermore, atovaquone suppressed cell proliferation and angiogenesis in vivo, promoted apoptosis, and prolonged the survival period of tumor-bearing mice, all without any noticeable negative consequences [210,211].

**Table 2:** Repurposed drugs for specific cancer type treatment with their chemical structure, old and new targets, and development status.

New Therapeutic Indication	Pharmacological Class	Drug Name	Chemical Structure	New Target	Original Target	Status	References
*Prostate Cancer*	*Anti-Dyslipidemic Drugs*	Atorvastatin (Statins)	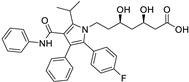	Block cholesterol synthesis and androgen production	HMG-CoA reductase	Phase II trials (NCT01821404)	[47,48,49,159,160]
	*Antiarrhythmic Drugs*	Digoxin	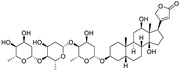	Cell cycle topoisomerase II	Na/K pump inhibition	Preclinical	[59,60,61,161,212]
		Ouabain	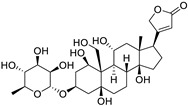	Apoptosis by blocking the production of survivin and STAT3	Na/K pump inhibition	Preclinical	[161,212]
	*Anti-Inflammatory Drugs*	Indomethacin	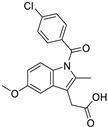	MYC gene family expression	Cyclooxygenases (COXs)	Preclinical	[179]
	*Antidepressant Drugs*	Sertraline	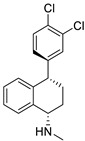	Angiogenesis, metastasis, and autophagy	Selective serotonin reuptake inhibitor	Preclinical	[180]
	*Beta-Blockers*	Propranolol	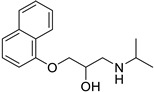	CREB1-EZH2-TSP1 pathway	Beta receptors	Phase II trials (NCT03152786)	[181]
	*Miscellaneous*	Heparin	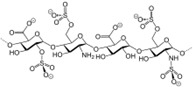	Cytokine, adhesion molecule, and angiogenic factor expression	Antithrombin	Preclinical	[182]
		Zoledronic acid	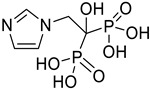	G1 arrest, metastasis	Osteoclast proliferation	Preclinical	[183,184]
		Mifepristone	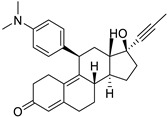	Suppressing over-expressed cell surface receptors in CRPC cells	Anti-progesterone	Preclinical	[183,184]
		Rapamycin	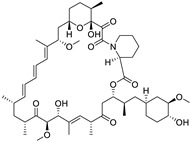	Binding to the FRB domain of mTOR, inhibiting mTORC1	Cytokine signaling	Preclinical	[50,51,165,166,167,183,184]
*Gastric cancer (GC)*	*Antidepressant*	Fluoxetine	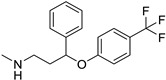	Inhibition of the endoplasmic reticulum stress marker (CHOP)	Serotonin reuptake inhibitor (SSRI)	Preclinical	[185]
		Paroxetine	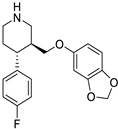	Enhanced DNA damage and reduced DNA repair		Preclinical	[185]
	*Anti-Epileptic*	Valproic acid (VPA)	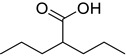	HDAC1/PTEN/Akt signaling pathway	Na channel	Phase II trials (NCT00496444)	[155,156,185,186]
	*Antipsychotic*	Risperidone	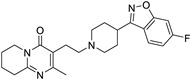	Caspase 3, 8,9, ROS	Serotonin and norepinephrine reuptake inhibition	Preclinical	[187,188]
	*Agents that chelate iron*	Deferasirox	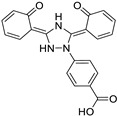	NDRG1, mTOR, and c-myc expression	Iron chelator/iron toxicity	Preclinical	[185,189]
*Blood malignancies*	*Antiemetic*	Thalidomide	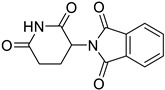	TNF-α	Immunomodulation	Approved	[190,191,192,193]
	*Tyrosine kinase inhibitor*	Imatinib	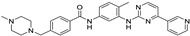	Suppression of KIT kinase gastrointestinal stromal tumors (GISTs)	Chronic myelogenous leukemia	Approved	[194,195,196,197]
		Dasatinib	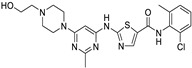	Philadelphia chromosome-positive acute lymphoblastic leukemia (ALL)	Chronic myeloid leukemia (CML)	Approved	[196,197]
*Breast cancer*	*Antibiotic*	Nitroxoline (NTX)	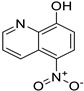	Anti-angiogenic activity, cathepsin B	Chelation of divalent cations	Preclinical	[157,200]
*Colon cancer*	*Antibiotic*	Rapamycin	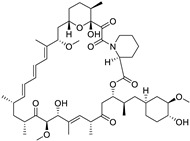	mTOR pathway	mTOR pathway	Preclinical	[50,51,165,166,167,183,184]
	*Anti-Helminthic*	Mebendazole (benzimidazole)	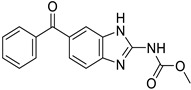	MYC pathway	Tubulin polymerization	Phase III trials (NCT03925662)	[62,70,172]
		Niclosamide	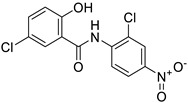		Wnt/β-catenin cascade	Phase I trials (NCT02687009)	[162,163,164]
*Hepatocellular carcinoma*	*Antipsychotic*	Pimozide	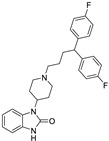	Apoptosis expression of STAT3	DRD2	Preclinical	[141,142,205,206,213,214]
	*Antiarrhythmic*	Amiodarone	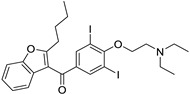	mTOR inhibitor	Na and K channels	Preclinical, meta-analysis	[207,208]
		Lanatoside C	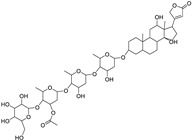	Mitochondrial membrane potential (MMP), AKT/mTOR, PKCd	Na channel	Preclinical	[207,209]
	*Anti-Microbe*	Atovaquone	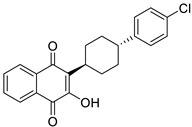	p53 and p21, kinase-2 and H2AX	ETC at the bc1 complex	Preclinical	[210,215,216]

### 2.3. Repurposing with a Mechanism-Directed Anticancer Activity

#### 2.3.1. Repurposed Medications to Target Mitochondrial Energy Metabolism in Cancer

- Anti-diabetic

Another notable characteristic of metformin is its capacity to specifically target oxidative phosphorylation. Empirical data indicates that metformin may exert its anti-diabetic properties by specifically targeting complex I of the mitochondrial respiratory chain. Indeed, recent studies have shown that metformin blunts the function of complex I in a wide range of cancers [60,168].

Moreover, metformin is believed to inhibit complex I, which is responsible for a reduction in oxygen consumption in various cancer models. Metformin also decreases ATP production in a variety of malignancies. In some instances, the expression levels of complex I transcripts were altered, and the NADH/NAD ratio was reduced. Recent research indicates that the metabolic milieu of the tumor is the determining factor in the antiproliferative effect of metformin, as it has the potential to significantly alter the sensitivity to the drug [60,168]. In general, metformin primarily affects cancer progression by increasing the rate of cell death and decreasing the proliferation of tumorous cells. Phenformin has been demonstrated to enhance the radiosensitivity of cancer cells and suppress complex I expression and oxygen consumption in CRC. Phenformin has been shown to inhibit tumor growth in mouse models of cancer with oxidative phosphorylation failure. Moreover, phenformin and the oxamate inhibitor of lactate dehydrogenase synergistically decrease respiration and ATP generation [169,170,217].

Similarly, this drug combination suppresses the proliferation of cells and the growth of malignancies. Canaglifozin, a pharmacological agent used to treat diabetes, suppresses the proliferation of complex I and carcinoma cells in prostate and lung cancer and exhibits a synergistic impact when combined with radiation and chemotherapy. Pioglitazione, when combined with the glycolysis inhibitor 2-deoxyglucose, produces an antiproliferative action, suppresses oxygen consumption, and specifically affects mitochondria [171,174,218,219].

- Anti-microbe

Indeed, adverse metabolic events (AMs) have the potential to cause mitochondrial malfunction in both healthy and cancerous cells. However, malignant cells that undergo rapid division and need a substantial energy input are particularly vulnerable to AMs. Additional comprehensive examples of antimicrobials that inhibit cancer by altering mitochondrial aerobic metabolism are presented below. Members of the tetracycline and chloramphenicol families of frequently used antibiotics block both mitochondrial and bacterial translation. According to the inhibition of mitochondrial translation, these antibiotics reduce the activities of oxidative phosphorylation complexes I, IV, and V, which consist of components encoded by mitochondria [174,175,176].

Originally designed to eliminate aerobic bacteria, the antibiotic bedaquiline specifically targets mitochondrial and bacterial ATP synthase [123]. The anti-malarial medication atovaquone acts as an inhibitor of the oxidative phosphorylation complex III. Ivermectin, a therapy that is particularly effective against a range of parasites, inhibits Complex I activity [215,216].

Pyrvinium pamoate prevents the activity of the mitochondrial NADH-fumarate reductase machinery, consisting of complex I and II, which is responsible for treating malaria and pinworm infections [220,221].

The broad-spectrum antifungal drug itraconazole binds to the voltage-dependent mitochondrial protein anion-selective channel 1 (VDAC1) and reduces ATP synthesis [222,223,224].

Furthermore, quinolones, aminoglycosides, β-lactams, and oxazolidinones are further categories of antibacterial substances that cause impairment of mitochondrial activity in mammalian cells. Nevertheless, the impact of their antibacterial properties on aerobic metabolism in cancer cells has not been thoroughly investigated [225,226].

#### 2.3.2. Repurposed Drugs as ROS Inducer in Cancer

Tigecycline is an antibiotic approved by the FDA for treating pneumonia, complex infections, and multidrug-resistant bacterial infections. It inhibits protein synthesis, leading to bacterial death. Tigecycline is highly potent against cancer by increasing reactive oxygen species levels. It suppresses mitochondrial respiration, membrane potential, and ATP levels, causing cell death. It also enhances the effectiveness of traditional cisplatin against human hepatocellular carcinoma. In addition, a Phase I clinical trial evaluating the safety and biological efficacy of intravenous tigecycline infusions for acute myeloid leukemia was successfully completed [177,227].

Levofloxacin, a third-generation fluoroquinolone antibiotic, has also shown potential for cancer treatment. It suppresses lung cancer cell proliferation and apoptosis by inhibiting mitochondrial electron transport chain complex function and ROS production. It selectively targets breast cancer cells and works with 5-FU to inhibit mitochondria production [178,227].

Albendazole is a non-toxic anti-parasitic medication that eliminates parasites by suppressing ATP synthesis and restricting glycogen reserves. It has been shown to induce oxidative stress in cancer treatment, triggering apoptosis and cell death in MCF-7 cells. This is linked to reduced glutathione levels, enrichment of oxidative biomarkers, and increased antioxidant enzyme activity [173,228].

Pimozide, an FDA-approved antipsychotic medication, was first identified as a dopamine antagonist with anti-melanoma cancer activity in 1979. It was tested in cancer cells, showing it suppressed cancer cells by generating ROS. However, clinical trials investigating its anticancer effects have not been conducted [141,205,213,214].

**Table 3:** Repurposed drugs for cancer treatment with directed mechanism with their chemical structure, old and new indications, and development status.

Targeted Mechanism	Pharmacological Class	Drug Name	Chemical Structure	New Therapeutic Indication	Development Status	References
*Mitochondrial energy metabolism*	*Anti-diabetic*	Metformin (biguanides)	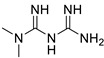	Colorectal, breast, pancreatic, prostate, lung, and cervical malignancies	Phase II (NCT01632020) and III trials (NCT05921942)	[60,168,169]
		Phenformin	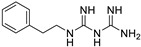	Colorectal cancer	Phase I trials (NCT03026517)	[169,170,217]
		Canagliflozin	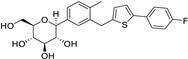	Prostate and lung cancer	Preclinical	[174,218,219]
		Pioglitazone	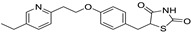	Breast, prostate, and lung cancer	Phase II trials (NCT00780234)	[55,56,57,58,59,60,61,218,219]
	*Anti-microbe*	Bedaquiline	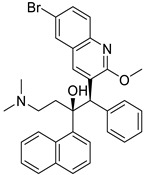	Breast	Preclinical	[123]
		Ivermectin	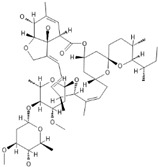	Pancreatic and colorectal cancers	Preclinical	[215,216]
		Pyrvinium	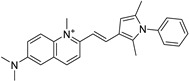	Pancreatic, colorectal, and breast cancers	Phase I (NCT05055323) trials	[220,221]
		Itraconazole	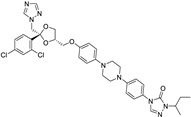	Non-small cell lung cancer (NSCLC), prostate cancer, and basal cell carcinoma (BCC)	Phase II (NCT00769600) trials	[222,223,224]
*ROS inducer*	*Antibiotic*	Tigecycline	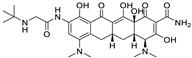	Hepatocellular carcinoma and acute myeloid leukemia	Phase I (NCT01332786) trials	[177,227]
		Levofloxacin	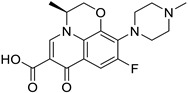	Lung and breast cancer	Preclinical	[178,227]
	*Anti-parasitic*	Albendazole	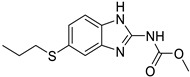	Ovarian, colorectal, and pancreatic cancers	Preclinical	[173,228]
	*Antipsychotic*	Pimozide	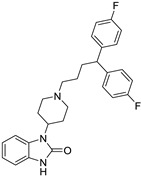	Hepatocellular carcinoma	Preclinical	[141,205,213,214]

## 3. Conclusions

The approach to cancer treatment known as drug repurposing is promising, as it provides a window of opportunity for drug discovery and is a developing trend in the field. Globally, cancer is one of the most prevalent causes of mortality, which has led to economic and public health concerns. Nevertheless, cancer treatment encounters numerous challenges, including the development of resistance mechanisms in tumor cells to anticancer agents and the reduction in the therapeutic efficacy of existing treatments. This underscores the pressing necessity for the creation of innovative medicines or strategies to combat cancer.

Drug repurposing is a cost-effective and time-saving method for enhancing the number of clinically available drugs to treat cancer, thereby contributing to the development of more personalized treatments. The current popular topic in cancer treatment is medicine of precision, which is a result of the heterogeneity associated with this disease.

Human malignancies are distinct maladies that necessitate distinct treatment options and approaches. In the past two decades, there has been substantial progress in the discovery of novel pharmaceuticals for a variety of malignant diseases. However, a considerable proportion of cancer patients are ultimately untreatable due to the development of resistance to existing drugs, which is a cause of exasperation for scientists and physicians. Many nations lack the financial capabilities to cover the escalating costs of contemporary chemotherapy treatment. Hence, drug repurposing is seen as a highly promising approach for the identification of novel anticancer treatments that are both economically efficient and can be promptly authorized for commercialization.

Clinical trials are currently being conducted with repurposed medications that showed promising anticancer activities in preclinical studies. Certain medications have been successfully repurposed and received FDA approval for the treatment of human malignant disorders, while others are still in the development phase. A pharmacological meta-analysis of drugs, including aspirin, statins, and metformin, found a significant association with a decreased risk of cancer. These medications are expected to progress further with clinical approval for cancer therapy.

The significance of drug repurposing as a strategy for the rapid and cost-effective identification of potential anticancer agents is now being increasingly supported by a growing body of evidence.

In summary, drug repurposing presents substantial opportunities in cancer therapy, enabling the development of more cost-effective and expedited treatment options. The development of innovative cancer therapies and the improvement of patient outcomes can be expedited by confronting the challenges encountered by repurposed medicines and capitalizing on their unique opportunities.

This raises the question of whether it is worthwhile to focus more on the clinical development of approved medications that can be used to treat cancer because of their proven effectiveness in preclinical and early clinical studies rather than new molecule discovery, which is more expensive and time-consuming.

## Data Availability

No new data were created or analyzed in this study.
